# Biotribological Tests of Osteochondral Grafts after Treatment with Pro-Inflammatory Cytokines

**DOI:** 10.1177/1947603521994900

**Published:** 2021-02-17

**Authors:** Christoph Bauer, Hakan Göçerler, Eugenia Niculescu-Morzsa, Vivek Jeyakumar, Christoph Stotter, Thomas Klestil, Friedrich Franek, Stefan Nehrer

**Affiliations:** 1 Department for Health Sciences, Medicine and Research, Center for Regenerative Medicine, Danube University Krems, Krems, Austria; 2 AC2T research GmbH, Wiener Neustadt, Austria; 3 LK Baden-Mödling-Hainburg, Department of Orthopedics and Traumatology, Baden, Austria; 4 Center for Medical Specializations, Department for Health Sciences, Medicine and Research, Danube University Krems, Krems, Austria

**Keywords:** osteoarthritis, cytokines, biotribology, friction, histology

## Abstract

**Objective:**

During osteoarthritis progression, cartilage degrades in a manner that influences its biomechanical and biotribological properties, while chondrocytes reduce the synthesis of extracellular matrix components and become apoptotic. This study investigates the effects of inflammation on cartilage under biomechanical stress using biotribological tests.

**Methods:**

Bovine osteochondral grafts from five animals were punched out from the medial condyle and treated with or without pro-inflammatory cytokines (interleukin-1β [IL-1β], tumor necrosis factor-α [TNF-α], IL-6) for 2 weeks. After incubation, biotribological tests were performed for 2 hours (alternating 10 minutes test and pause respectively at 39°C, 180 N, 1 Hz, and 2 mm stroke). Before and after testing, the cartilage surface was imaged with a 3-dimensional microscope. During testing, the coefficient of friction (COF) was measured, while gene expression analysis and investigation of metabolic activity of chondrocytes were carried out after testing. Histological sections of the tissue and wear debris from the test fluid were also analyzed.

**Results:**

After biotribological tests, surface cracks were found in both treated and untreated osteochondral grafts. In treated grafts, the COF increased, and the proteoglycan content in the cartilage tissue decreased, leading to structural changes. Chondrocytes from treated grafts showed increased expression of genes encoding for degradative enzymes, while cartilage-specific gene expression and metabolic activity exhibited no significant differences between treated and untreated groups. No measurable difference in the wear debris in the test fluid was found.

**Conclusions:**

Treatment of osteochondral grafts with cytokines results in a significantly increased COF, while also leading to significant changes in cartilage proteoglycan content and cartilage matrix compression during biotribological tests.

## Introduction

Osteoarthritis (OA) is one of the most common degenerative joint diseases worldwide, and frequently affects the hands and weightbearing joints of the body.^1,2^ Etiological causes of the disease are diverse. Most common are biochemical imbalances between anabolic and catabolic factors as well as progressive surface degradation caused by mechanical stress.^3^ The pathogenesis of OA also leads to the formation of osteophytes, remodeling of the subchondral bone, and inflammation in the joint.^4,5^ This inflammation is characterized by the release of pro-inflammatory cytokines such as interleukin-1β (IL-1β), tumor necrosis factor-α (TNF-α), and IL-6 as well as proteolytic mediators like matrix metalloproteinases (MMPs).^6-8^ All these factors lead to an imbalance in metabolic homeostasis of the cartilage tissue followed by degradation and alteration of the synovial fluid. As a consequence, the biomechanical and biotribological properties of the joint are influenced,^9,10^ which can lead to chondrocyte apoptosis and reduced synthesis of important components of the extracellular matrix (ECM).

The lubrication of synovial joints involves a complex interaction between different factors such as tissue composition, structure, and mechanics. The excellent friction and wear properties of articular cartilage are achieved by a mixed lubrication regime that includes fluid-film lubrication by synovial fluid and boundary lubrication by thin films on the cartilage surfaces.^11^ These properties can be disturbed and negatively affected by inflammation processes such as those occurring in OA. Increased friction in osteoarthritic joints is attributed to decreasing load support of interstitial fluid, which can be squeezed out with the progression of OA, and the altered rheological properties of the synovial fluid.^12-14^ In the latter case, the reduced capacity for boundary lubrication, which is associated with a decreased level of lubricin, plays an essential role.^15,16^ This further increases the risk of joint damage and the progression of OA.^16,17^

Various studies have used degradative enzymes to examine the biomechanical and biotribological properties of cartilage, investigating changes in the mechanism of fluid support as well as depletion of glycosaminoglycans from the cartilage matrix.^13,18^ Both led to an increased coefficient of friction.^13,19^ Most tests involved the use of a pin-on-disc tribometer, where the cartilage was not moved against cartilage tissue; instead, other materials—such as metal, ceramic, or glass—were used. However, the natural response of the tissue due to movement and stress on other soft tissue such as cartilage has typically been neglected.^20-22^ Understanding the change in the frictional properties of cartilage as well as the role of inflammation in this process may lead to new means of suppressing joint degradation.

The aim of this study was to investigate the influence of pro-inflammatory cytokines on osteochondral grafts in a well-established *ex vivo* test system with biotribological and biological outcome measures. In our experimental setup, both untreated and treated surfaces of osteochondral grafts were slid over one another. Our hypothesis was that treatment with pro-inflammatory cytokines would result in increased surface damage, a higher coefficient of friction, and reduced cartilage-specific parameters (e.g., gene expression, metabolic activity) compared to untreated osteochondral grafts in a cartilage-on-cartilage biotribological test system.

## Methods

### Specimen Preparation

Five bovine knees were obtained from cows slaughtered between the ages of 18 and 20 months. Under aseptic conditions, osteochondral grafts were harvested from the medial femoral condyle using a Single-Use OATS punch (Arthrex Inc., Naples, FL, USA). Each knee yielded 12 to 16 osteochondral grafts (8 mm diameter, 15 mm height). The osteochondral grafts were washed for 2 hours in phosphate-buffered saline (PBS, Sigma-Aldrich Chemie GmbH, Steinheim, Germany) at 37°C to remove loose bone particles and fatty tissue. The samples were then cut to 8 mm height with a custom-made cartilage holder.

### Grouping of the Cartilage Grafts

In total, 4 groups of osteochondral grafts (**Table 1**) were used in this study with 3 osteochondral grafts in each group (2 grafts for metabolic activity and gene expression; 1 graft for histology).

**Table 1.** Classification of the Test Groups.

		Untested	Biotribologically Tested
**Untreated**	Group 1	×	
	Group 2		×
**Cytokine-treated**	Group 3	×	
	Group 4		×

### Treatment with Pro-Inflammatory Mediators

Each osteochondral sample of the control and treatment group were cultivated for 2 weeks in 3 mL growth medium (GIBCO DMEM/F12 GlutaMAX-I, Life Technologies, Carlsbad, CA, USA) supplemented with 5% fetal calf serum (FCS; GIBCO, Life Technologies), antibiotics (penicillin 200 U/mL; streptomycin 0.2 mg/mL), amphotericin B 2.5 µg/mL (Sigma-Aldrich Chemie GmbH, Steinheim, Germany), and 0.05 mg/mL ascorbic acid (Sigma-Aldrich Chemie GmbH, Steinheim, Germany). Culture medium was changed every 3 days. In the treatment group, the medium was additionally supplemented with the pro-inflammatory cytokines IL-1β, IL-6 and TNF-α (all 3 were used in a concentration of 10 ng/mL) (Sigma-Aldrich). After 2 weeks of incubation, biotribological tests for each animal were performed within the next 2 days at 39°C. During the testing time (2 hours), the untested group was also kept at 39°C. Before and after testing, the samples were stored at 4°C until both tested and untested osteochondral grafts were analyzed on day 17.

### Biotribological Test System

**Figure 1.**
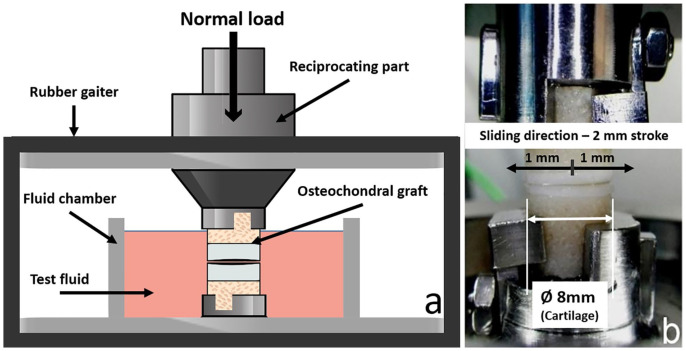
Biotribological test system. (**a**) Schematic of the designed biotribological installation, which was a pin-on-pin setup. (**b**) Image of the cartilage-against-cartilage contact in the biotribo-test system; upper graft holder is reciprocating during the tests with cytokine-treated or untreated osteochondral grafts.

A test system with a specially designed sample holder, which was already applied in previous studies,^23,24^ was used and is shown in **Fig. 1**. It performs a reciprocal sliding movement between the osteochondral grafts. These grafts are submerged in a test fluid (in this case PBS) to mimic conditions in the knee joint. The test setup itself encloses a sample holder to ensure sterile conditions throughout the testing process. This is necessary as biological samples should not be exposed to external influences, which may introduce artefacts and interfere in the process of further analysis.

The applied load was 180 N and, considering the contact area of 50.26 mm^2^ for 8 mm diameter cartilage samples, an initial estimated average pressure value of 3.57 MPa was achieved. The plane was assumed flat for all tested cartilage samples. The created tribo-model was a match in terms of contact pressure measurements for the tibiofemoral compartment of a human knee under the normal load of body weight with 0° of flexion.^25^ To mimic loading and unloading conditions of the knee during walking, the system was loaded for 10 minutes and then unloaded for another 10 minutes for a total test period of 2 hours (6 cycles) with a stroke of 2 mm and a frequency of 1 Hz.

For transportation purposes, samples that were initially stored at 4°C were equilibrated to room temperature for half an hour, followed by another half an hour in the specimen holder, and then submerged in PBS at a temperature of 39°C (internal bovine body temperature).

### Metabolic Activity

Metabolic activity of chondrocytes within the tissue was measured using an XTT-based *ex vivo* toxicology assay kit according to the manufacturer’s instructions (Cell Proliferation Kit II, Roche Diagnostics, Basel, Switzerland).

Cartilage was cut from the osteochondral grafts with a scalpel and divided longitudinally into two parts for XTT assay and RNA isolation. The cartilage was minced into smaller fragments on a 24-well plate. After tissue weight for each sample was determined, the tissue was incubated in the XTT solution (1 mL medium, 490 µL XTT reagent, and 10 µL activation reagent) for 4 hours at 37°C; the surrounding air contained 5% (v/v) CO_2_. After incubation, the XTT solution was removed and retained. Remaining tetrazolium product in the tissue was extracted by incubation with 0.5 mL dimethyl sulfoxide (DMSO) for 1 hour at room temperature under continual agitation. Then the XTT and DMSO solutions were pooled, and the absorbance was measured at 492 nm and 690 nm (background wavelength) in triplicates in a 96-well plate using a multimode microplate reader (Synergy 2, Winooski, VT, USA) with Gen 5 software. Absorbance was normalized to the wet weight of the tissue.

### RNA Isolation

The other half of the cartilage tissue retrieved from the osteochondral grafts was stored in RNAlater (Qiagen, Hilden, Germany) at 4°C for up to 1 week. After storage, the cartilage was minced into smaller fragments and transferred into tubes containing ceramic beads (MagNA Lyser Green Beads, Roche Diagnostics, Basel, Switzerland) with a 300 µL lysis buffer (10 µL β-mercaptoethanol + 290 µL RLT [from Fibrous Tissue Kit, Qiagen, Hilden, Germany]). Until RNA isolation, the tube was stored in liquid nitrogen. For RNA isolation, the tube was thawed and transferred to the MagNA Lyser (Roche Diagnostics, Basel, Switzerland) for homogenization of the cartilage tissue. The homogenization step (6,500 rpm, 20 seconds) was repeated 4 times with a 2-minute cooling phase after each step. According to the manufacturer’s instruction, every sample was then incubated with 20 µL proteinase K (from Fibrous Tissue Kit) for 30 minutes for a higher yield. RNA was eluted in 30 µL and stored at −80°C until cDNA synthesis.

### Gene Expression Analysis

Gene expression analysis was carried out as previously described.^26^ Briefly, cDNA synthesis was performed using Transcriptor First Strand cDNA Synthesis Kit (Roche, Basel, Switzerland). Additionally, RNA from bacteriophage MS2 was added to stabilize the isolated RNA during cDNA synthesis. Real-time quantitative polymerase chain reaction (RT-qPCR) was performed in triplicate using the LightCycler 96 from Roche (Basel, Switzerland). In total, 4 genes—collagen type 2 (COL2A1), aggrecan (ACAN), matrix metalloproteinase-1 (MMP1), and matrix metalloproteinase-13 (MMP13)—were analyzed, while glyceraldehyde-3-phosphate dehydrogenase (GAPDH) was used as housekeeping gene (**Table 2**).

**Table 2.** Sequences of Primers and Conditions Used in Quantitative Polymerase Chain Reaction.

Primer	Abbreviation	Sequence (3′–5′)
**Glyceraldehyde-3-phophate dehydrogenase**	GAPDH	
**Forward**		ATGTTCCAGTATGATTCCACCC
**Probe**		AGCTTCCCGTTCTCTGCCTTGAC
**Reverse**		ATACTCAGCACCAGCATCAC
**Aggrecan core protein 1**	ACAN	
**Forward**		ACCTACGATGTCTACTGCTACG
**Probe**		AGAAGGTGAACTGCTCCAGGCG
**Reverse**		AGAGTGGCGTTTTGGGATTC
**Collagen, type II, alpha 1**	COL2A1	
**Forward**		GTGCAACTGGTCCTCTGG
**Probe**		CCTTGTTCGCCTTTGAAGCCAGC
**Reverse**		ACCTCTTTTCCCTTCTTCACC
**Matrix metalloproteinase 1**	MMP1	
**Forward**		TTCAACCAGGTGCAGGTATC
**Probe**		AAATTCATGCGCTGCCACCCG
**Reverse**		AGCCCCAATGTCAGTAGAATG
**Matrix metalloproteinase 13**	MMP13	
**Forward**		CTAAACATCCCAAAACGCCAG
**Probe**		CCCTTGATGCCATAACCAGTCTCCG
**Reverse**		ACAGCTCTGCTTCAACCTG

### Histology

For histological analysis, osteochondral grafts were fixed in 4% buffered formaldehyde solution (VWR, Radnor, PA, USA) for up to 1 week and decalcified under constant agitation using Osteosoft solution (Merck, Burlington, MA, USA). After decalcification (duration of 4-6 weeks), the osteochondral grafts were embedded in Tissue-Tek OCT (optimal cutting temperature, VWR, Radnor, PA, USA) and stored at −80°C. Sectioning was done using the CryoStar NX70 Cryostat (Thermo Fischer Scientific, Waltham, MA, USA), with −25°C for the knife temperature and −20°C for the chamber temperature. Six-micrometer sections were obtained and processed for safranin O/light green staining. Images were taken with a Leica DM-1000 microscope and processed using the Leica Manager software (Leica, Wetzlar, Germany). To quantify changes within the cartilage sections stained with safranin O/light green, a modified Mankin scoring system was used (**Table 3**).^27^ The assessment was done by 5 independent observers with a maximum score of 15.

**Table 3.** Components of the Modified Mankin Scoring System.

Structure	Cellularity	Matrix Staining	Tidemark Integrity	Score
**Smooth surface/normal**	Normal arrangement	Normal staining	Normal and intact	0
**Roughened surface/single crack or area of delamination**	Clustering in superficial layer or loss of cells up to 10%	Slight loss of stain	Disrupted	1
**Multiple cracks/moderate delamination**	Disorganization or loss up to 25%	Moderate loss of stain	×	2
**Fragmentation in cartilage or severe delamination**	Cell rows absent or loss up to 50%	Severe loss of stain	×	3
**Loss of fragments**	Very few cells present	No stain present	×	4
**Complete erosion to tidemark**	×	×	×	5
**Erosion beyond tidemark**	×	×	×	6

### Microscopic Images

The InfiniteFocus G5 3D microscope (Alicona Imaging GmbH, Graz, Austria) was used to optically analyze the cartilage surface before and after the test to reveal determinant surface roughness parameters for the biotribological performance of cartilage tissues. PBS was added every 5 minutes to prevent the cartilage surface from drying out. The InfiniteFocus G5 uses Focus Variation technology, combining the small depth of focus of an optical system with vertical scanning to provide topographical and color information from the variation of focus. Due to the vertical movement of the precision optics along the optical axis with continuously capturing data from the surface, each region of the object can be sharply focused. Algorithms convert the acquired sensor data into 3-dimensional information and a true color image with full depth of field. This is achieved by analyzing the variation of focus along the vertical axis. The initial image of the cartilage was further processed to obtain a surface profile. This method was described in our previous study in detail.^23^

### Sulfated Glycosaminoglycans (sGAG)

The quantification of sGAG was performed according to Barbosa *et al*.^28^ In brief, fluid (PBS) used during biotribological tests was treated overnight with 25 U/mL proteinase K (Sigma-Aldrich, St. Louis, MO, USA) at 56°C. After inactivation of the enzyme (90°C, 10 minutes), the fluid was collected in ultra-free filter reaction tubes of 0.1 µm pore size (Millipore, Burlington, MA, USA) and centrifuged (12,000*g*, 4 minutes, room temperature). One milliliter of a 1,9-dimethyl-methylene blue solution (DMMB) was added to 100 µL filtrate and vigorously mixed to allow the formation of complexes of DMMB and sGAG in the sample. The complexes were pelleted via centrifugation (12,000*g*, 10 minutes, room temperature) and subsequently dissolved in a decomplexation solution. After 30 minutes of shaking, the absorbance was measured at 656 nm photometrically using an Ultrospec 3300 pro spectrophotometer (Amersham Bioscience plc, Amersham, UK). The sGAG amount was calculated from a standard curve with shark chondroitin sulphate (Sigma-Aldrich, St. Louis, MO, USA). The measurement for both treated and untreated posttesting osteochondral grafts was performed in duplicate.

### Hydroxyproline (HYP) Assay

Total collagen content was determined by quantifying the hydroxyproline content. Test fluid, after biotribological tests, was hydrolysed in 6 M HCl at 110°C for 18 hours and the hydroxyproline content from the hydrolyzed solution was measured with a chloramine-T/Ehrlich spectrophotometry-based assay at a wavelength of 560 nm.

### Statistical Analysis

All statistical analysis was performed using GraphPad Prism Software (GraphPad Prism Software Inc., San Diego, CA, USA). The statistical analysis was carried out using a 1-way analysis of variance. Multiple comparisons were performed via a nonparametric Kruskal-Wallis test followed by Dunn’s post hoc test. Data from the metabolic activity and gene expression are shown in a box plot to represent median, first quartile, and third quartile, with error bars indicating maximum and minimum values. Where outliers were present, dots above or below are shown. The values for the coefficient of friction are reported as means ± standard error of the mean (means ± SEM). Statistical significance was set at *P* < 0.05.

## Results

### Osteochondral Grafts

Punched out osteochondral grafts for biotribological tests had a symmetrical flat surface in almost every sample used for the experiments. Asymmetric grafts, which were not possible to avoid, were used for the untested control and treatment group as flatness was not a critical factor as it was for grafts used in biotribological tests.

### Metabolic Activity of the Cells

**Figure 2.**
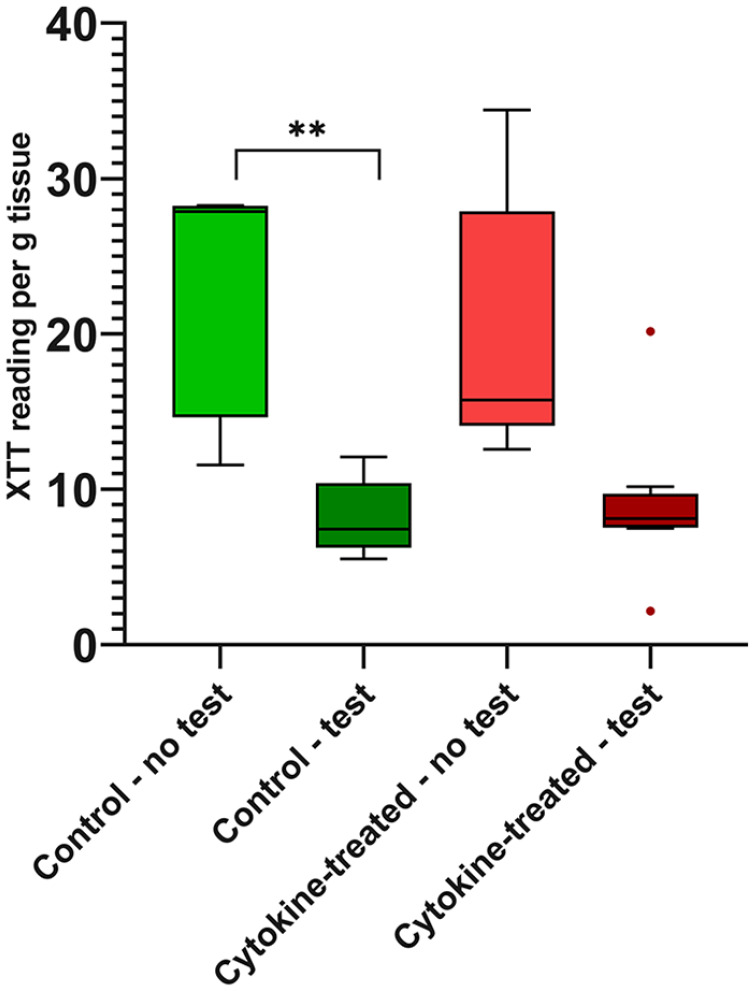
Measurement of the metabolic activity. Chondrocytes from tested and untested osteochondral grafts after treatment with and without pro-inflammatory cytokines were analyzed concerning their metabolic activity using XTT assay. *n* = 5 animals/group; **P < 0.01. Data are expressed as median and range with Tukey box-and-whisker plot: lower box = 75 percentile, upper box = 25 percentile, whisker = nonoutlier range, dot = outlier (>1.5-fold above/below the box).

The metabolic activity of chondrocytes in osteochondral grafts showed no differences between the untested (control and cytokine-treated) groups. Metabolic activity of the tested control group was significantly lower (4-fold of the median value) than the untested control group (**Fig. 2**). Cytokine treatment of osteochondral grafts showed similar results (2-fold decrease of the median value) with some outliers in the tested group, resulting in a nonsignificant difference.

### Expression of Anabolic and Catabolic Cartilage-Specific Genes

**Figure 3.**
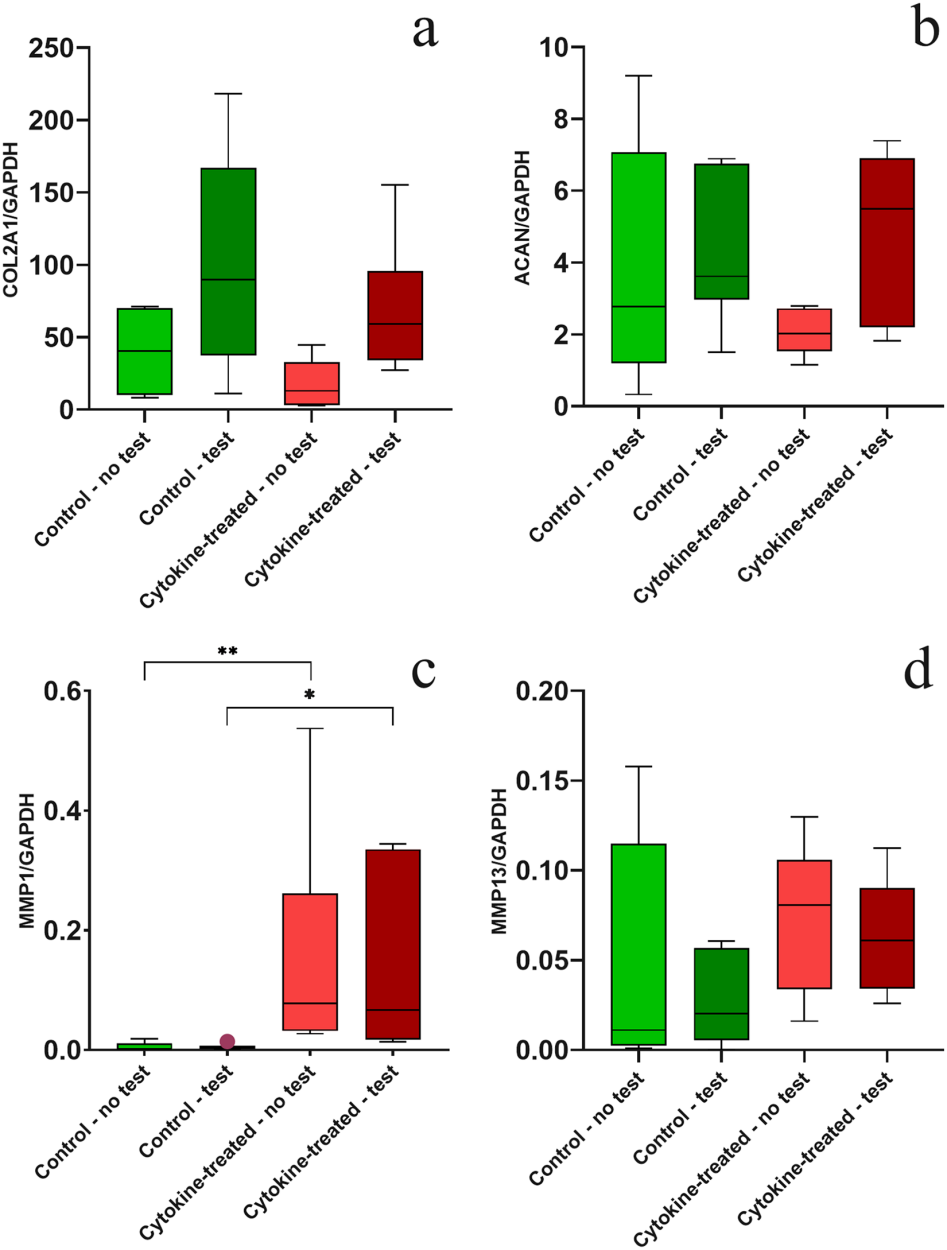
Gene expression analysis of (**a**) COL2A1, (**b**) ACAN, (**c**) MMP1, and (**d**) MMP13 in biotribologically tested and untested osteochondral grafts with and without pro-inflammatory cytokine treatment. *n* = 5 animals/group; *P < 0.05, **P < 0.001. Data are expressed as median and range with Tukey box-and-whisker plot: lower box = 75 percentile, upper box = 25 percentile, whisker = nonoutlier range, dot = outlier (>1.5-fold above/below the box).

For the analysis of gene expression, specific bovine primers were designed and tested successfully regarding optimal temperature values for primer annealing. After 17 days, the anabolic marker COL2A1 showed a nonsignificant difference in the untested groups, whereas the expression of osteochondral grafts treated with pro-inflammatory cytokines tended to be decreased (**Fig. 3a**). After the biotribological tests, an approximately 2-fold increase in median value could be observed in both groups when compared to the untested groups, but this was not statistically significant. The analysis of ACAN (**Fig. 3b**), another anabolic marker, showed a very similar expression pattern. Here, the treatment with cytokines led to a reduction of ACAN gene expression in the untested group and tended to be increased in a nonsignificant manner after the test. Gene expression values of the control groups were on the same level before and after testing with no statistical significance in comparison to the treatment groups.

In addition, **Figure 3** shows the expression of the catabolic genes MMP1 (**Fig. 3c**) and MMP13 (**Fig. 3d**), 2 genes involved in the breakdown of interstitial collagens (e.g., types I, II, and III). The occurrence of pro-inflammatory cytokines led to a significantly increased expression of MMP1 in the untested and tested group compared with both control groups (tested and untested), where levels were near the detection limit. The gene MMP13 was also highly expressed with cytokine treatment, to the degree that a significant difference could not be shown between treated and untreated groups. After biotribological tests, the expression of MMP13 remained on a constant level when comparing tested versus untested groups.

### Microscope Images

**Figure 4.**
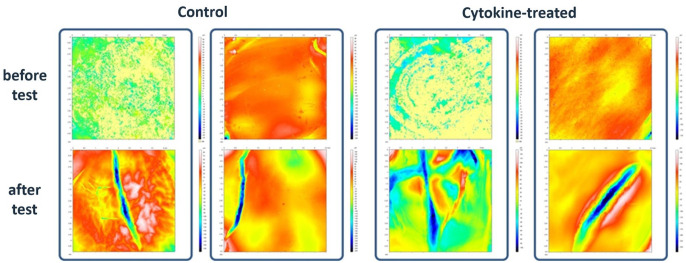
Alicona 3-dimensional microscope images. Representing images of major changes on the cartilage surface structure before and after biotribological tests for untreated and cytokine-treated osteochondral grafts.

Prior to biotribological tests, microscopic images of the surfaces of untreated and treated osteochondral grafts showed no cracks and fissures (**Fig. 4**) or other microscopically visible damages. Only height differences of up to 90 µm in the starting material were observed. However, our focus was on the formation of superficial cracks and fissures, which are caused by the applied load under physiological conditions (3.57 MPa). **Fig. 4** also shows that the specifically applied load causes cracks and fissures in cartilage tissue, but without differences between cytokine-treated and untreated osteochondral grafts. The extent of surface changes ranged from deep trenches (up to 250 µm) to small superficial cracks (up to 20 µm). Since all grafts examined showed similar damage after biotribological tests, it was impossible to define a difference in cartilage surface between untreated and treated groups.

### Histology

**Figure 5.**
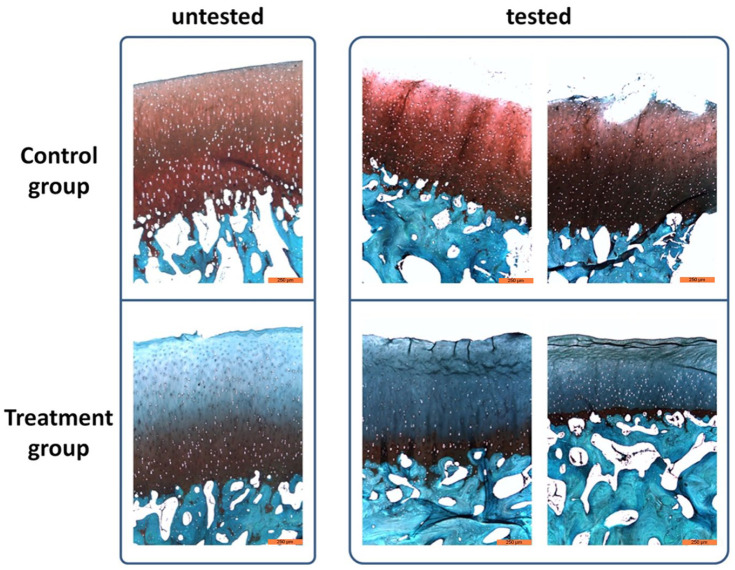
Histological assessment of osteochondral grafts. Exemplary histological images of cytokine-treated and untreated grafts in a biotribologically tested and untested state stained with safranin O/light green. Scale bar 250 µm.

For histological analysis, safranin O/light green staining was performed as shown in **Fig. 5** and used as an additional means of finding any differences (e.g., cracks or fissures) between untreated and treated osteochondral grafts in a biotribologically tested and untested state.

Osteochondral grafts in the control group retained a higher amount of proteoglycans in the untested state, which could slightly differ from graft to graft as a result of different harvesting locations. In comparison, proteoglycan content in the untested treatment group was highly reduced. Here, safranin O only stained proteoglycans in deeper layers of the cartilage, which are rich in proteoglycans. As a result, much higher differences between the control and treatment group were shown after the biotribological tests. In both biotribologically tested groups, cracks and fissures appeared on the cartilage surface as observed in microscopic images. In the control group, proteoglycan content was not influenced as the detected staining intensity was similar to the untested samples. Osteochondral grafts of the treatment group demonstrated a further reduced proteoglycan content in the tested state in comparison with the untested samples, indicating an influence by biotribological tests. Furthermore, histological sections not only exhibited superficial changes with cracks and fissures, but also a compression of the cartilage tissue itself. Here, pro-inflammatory cytokine treatment of osteochondral grafts aggravates the extrusion of interstitial fluid during mechanical loading. In addition to the vertical cracks, horizontal cracks appeared within the cartilage tissue. The evaluation of the histological sections using a modified Mankin Scoring System showed the qualitative differences quantitatively. In the untested control, the 5 observers agreed and rated it with 0 points. In comparison, the samples in an untested state and treated with cytokines achieved a mean value of 4.4 points. The biotribologically tested osteochondral grafts received a mean score of 3.2 points in the untreated state, while the cytokine-treated and -tested samples achieved a mean score of 8.

### Coefficient of Friction

**Figure 6.**
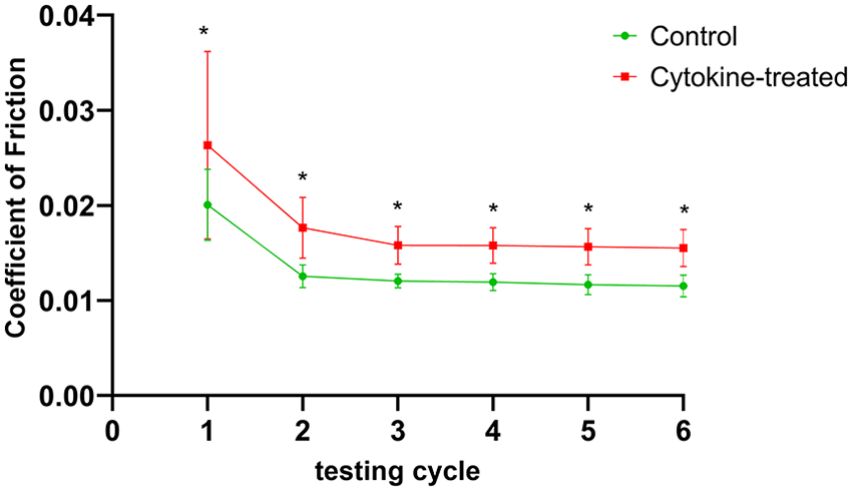
Coefficient of friction. During biotribological tests, the coefficient of friction was measured continuously; Calculation of mean values of untreated (green) and cytokine-treated (red) osteochondral grafts was performed from each test cycle (10 minutes of testing) of a total test period of 2 hours. *n* = 5 animals/group; *P < 0.05.

During biotribological tests, the coefficient of friction (COF) was recorded in each of the 6 cycles with significantly higher values measured in the first cycle in both the control (0.0200) and treatment group (0.0263) compared with subsequent cycles (**Fig. 6**). During the course of two-hour testing, the COF values for both groups reached a corresponding level from the second test cycle on. The control group values ranged between 0.0115 and 0.0125, while treatment group values were within a range of 0.0155 to 0.0176. Comparison of both groups showed a significant difference in COF for each test cycle.

### sGAG and HYP in the Supernatant after Testing

**Figure 7.**
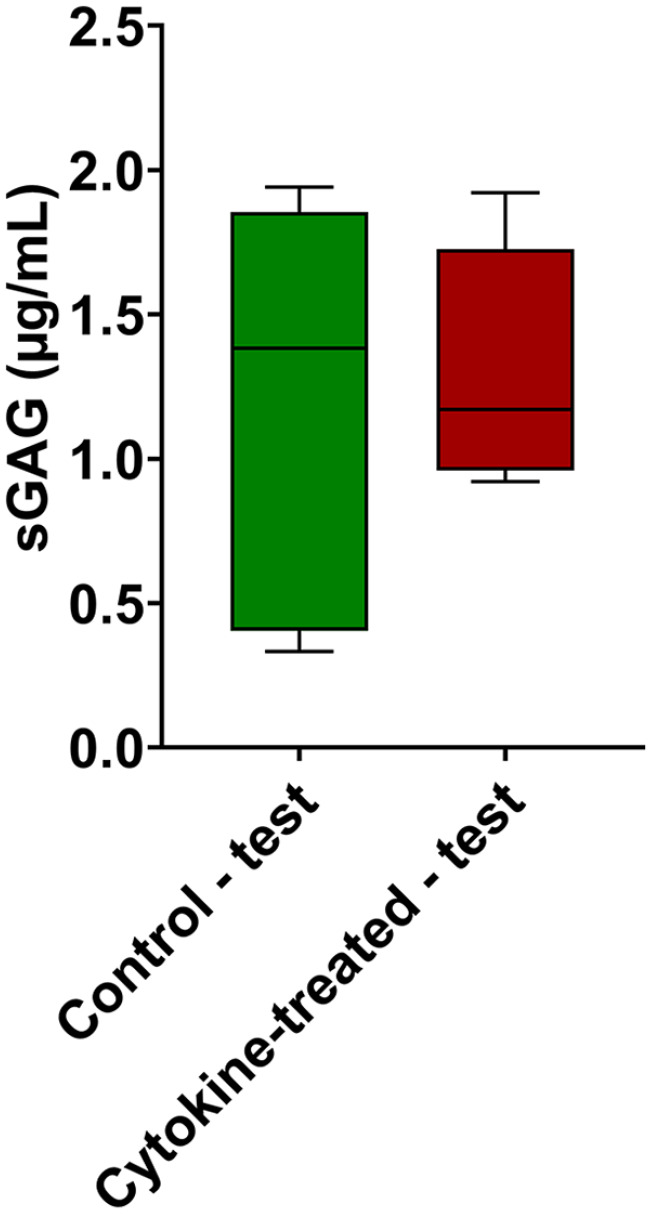
Quantification of sulfated glycosaminoglycans (sGAG). sGAG concentrations measured in the collected test fluids of cytokine-treated and untreated osteochondral grafts after biotribological tests. *n* = 5 animals/group. Data are expressed as median and range with Tukey box-and-whisker plot: lower box = 75 percentile, upper box = 25 percentile, whisker = nonoutlier range.

After biotribological testing of osteochondral grafts (control and treatment group), the test fluids (PBS) were collected and measured for their content of released or abraded sulfated glycosaminoglycans (sGAG), as well as for their hydroxyproline (HYP) content. There was no significant difference in sGAG content between treated and untreated osteochondral grafts in our test setup. Both groups in **Fig. 7** showed similar values for the respective test fluids after biotribological tests. In addition, hydroxyproline could not be detected in the test fluids of either group (no figure is shown).

## Discussion

The aim of this study was to identify possible differences between biotribologically tested and untested osteochondral grafts pretreated with and without pro-inflammatory cytokines. The grafts were taken from bovine cartilage of the medial-femoral condyle for reproducibility and availability, treated with cytokines and tested in an established cartilage-on-cartilage biotribological test system. In our experiments, we were able to show that treatment with pro-inflammatory cytokines leads to changes in the proteoglycan content of the cartilage, which is an already known phenomenon.^29^ Additionally, the applied force during biotribological tests leads to a compression of the cartilage matrix of treated osteochondral grafts. This compression could be the reason for the higher coefficient of friction measured in treated osteochondral grafts. The metabolic activity of the cells was only influenced by the biotribological test itself, while the gene expression of cartilage-specific and catabolic markers showed only small differences between the treated and untreated groups. No differences were observed in microscopic images of the cartilage surface after testing, except for cracks appearing in both test groups.

As shown in our previous study,^23^ the use of a cartilage-on-cartilage test system reflects native physiological conditions more accurately than testing against a metal or glass counterpart. This test setup is particularly advantageous when imitating inflammatory reactions, as this approach not only depends on the test fluid but also on how the cartilage matrix of both osteochondral grafts changes during treatment. The fact that the test system used here has a normal force of 180 N and thus exerts a pressure of 3.57 MPa on the cartilage surface of the osteochondral grafts ensures constant contact between the cartilage surfaces. The pressure achieved is comparable to pressure measurements of the tibial-femoral compartment in a human knee joint under normal physical load.^25^

The choice of cytokines in our study was limited to IL-1β, TNF-α, and IL-6, which are the most studied cytokines in relation to OA.^30^ IL-1β and TNF-α are the most important pro-inflammatory cytokines in this context, playing an important role in the pathogenesis and progression of the disease, while IL-6, which is secreted by chondrocytes especially when exposed to a stimulus like IL-1β,^31,32^ is considered a potential biomarker for early OA. These cytokines are first produced in the tissue and then released into the synovial fluid, resulting in the promotion of catabolic processes and enzymatic cartilage degradation.^33^ This is in accordance with findings in patients suffering from knee OA, where pro-inflammatory cytokines were detected in synovial fluid, cartilage and synovial membranes.^34-36^ The cytokine concentrations of 10 ng/mL used in this study are much higher than those found in synovial fluids taken from OA patients.^37,38^ However, these high concentrations were used to achieve a rapid effect on the cartilage tissue, which was reached after a two week period of incubation of the osteochondral grafts. This effect was confirmed by the strongly reduced proteoglycan content of the cartilage tissue shown in histological observations. Although this study only focused on this limited array of cytokines, it should be mentioned that there are other pro-inflammatory factors in the synovial fluid of patients. Though they are typically present in smaller quantities, their effect on osteochondral grafts could still be of relevance.

During biotribological tests, the coefficient of friction was measured as an indicator of mechanical stress. In the healthy knee joint, this unitless number is around 0.005.^39^ In our tests, this value could not be achieved, probably due to PBS test fluid being used as a substitute for synovial fluid. The values for the untreated osteochondral grafts varied between 0.0115 and 0.0125, which is comparable to lower levels measured in untreated grafts in our previous study.^23^ However, treatment with pro-inflammatory cytokines increased the coefficient of friction, with values measured between 0.0155 and 0.0176. This increase can probably be explained by the fact that the biotribological tests compressed the cartilage of the treated grafts and caused unevenness on the surface.

The surface of the osteochondral grafts was examined both before and after biotribological tests using an optical microscope. Prior to the tests, the surface of the untreated and treated group exhibited no damage, which indicates that the cartilage surface is not damaged during harvesting of the osteochondral grafts. After biotribological tests, cracks with a depth of up to 250 µm appeared in both treated and untreated groups. The shape and appearance of these surface cracks are comparable to other studies,^40,41^ including our previous study.^23^ For an additional assessment of the surface and extracellular matrix components of the cartilage tissue, histological sections of the osteochondral grafts were stained with safranin O/light green. These tests revealed that the proteoglycan content was significantly decreased in osteochondral grafts treated with pro-inflammatory cytokines. Similar results were shown by Gitelis *et al*.,^42^ when the cartilage tissue was incubated in a pro-inflammatory environment. Furthermore, histological images from treated grafts showed a level of proteoglycans comparable to Mankin Osteoarthritis Score 2 or OARSI (Osteoarthritis Research Society International) grade 2 to 3.^43,44^ This indicates that our treatment with cytokines had an effect comparable to a grade of degenerative OA. Subsequent biotribological tests in treated osteochondral grafts led to compression of the cartilage matrix with vertical tears in the tissue. In untreated samples, these changes did not occur. The altered biomechanical properties of the tested cartilage can thus be attributed to the loss of proteoglycans, as there was a decrease in compressive modulus of cartilage while the tissue was exposed to higher loads while being subjected to mechanical stress.^29^

Adding pro-inflammatory cytokines to the culture medium of osteochondral grafts reduced the expression of cartilage-specific genes such as collagen type 2 and aggrecan. It is well established that the cytokines used in this study reduce or inhibit the synthesis of these genes.^37,45,46^ The mechanical stress applied during biotribological tests reversed this reduction and also led to an increased expression in the untreated samples, especially the expression of collagen type 2. That mechanical stimulus can have such an effect has been demonstrated by several studies.^47,48^ Similarly, the expression of degradative enzymes was increased by pro-inflammatory cytokines, which further confirms that the culture conditions created for this study were similar to those found in OA. An increase of MMPs gene expression in bovine chondrocytes was also shown by Lv *et al*.^49^ when using IL-1β for the treatment of cartilage. In addition, this study was able to show that a dynamic mechanical stress in the physiological range does not lead to a difference in gene expression of MMPs.^49^ This was also confirmed by Fehrenbacher *et al*.,^50^ where a change in MMPs gene expression only occurs from about 12 MPa upward. The pressure of 3.57 MPa used in our study is within the physiological range and consequently had no effect on the gene expression of MMPs.

However, there are also some limitations to the current study that have to be taken into account. The experimental setup is an *ex vivo* model, which is difficult to compare with physiological conditions as there may occur abnormal loading conditions due to the fact that surrounding tissue is lacking. In addition, no strong conclusions regarding the biological and biomechanical properties of human cartilage can be made based on experiments with bovine cartilage. A further limitation is the use of 3 cytokines, while the synovial fluid of an OA patient contains many other pro-inflammatory and anti-inflammatory mediators. Additionally, under real-world conditions, damage to cartilage would usually occur over a longer duration. Here, only short-term effects on cartilage tissue are shown. Another limitation lies in the fact that the PBS test fluid used in this study is not comparable to synovial fluid, which also contains nutrients and lubricants (e.g., hyaluronic acid). Consequently, only a limited evaluation of cartilage metabolism based on the tested conditions is possible.

In conclusion, the present study shows that the treatment of osteochondral grafts with pro-inflammatory cytokines leads to a decrease in proteoglycan levels in the treated cartilage, which is comparable to progressive degenerative OA. Under a physiological load the decrease leads to changes in the biomechanical and biotribological properties of the cartilage with significant changes in tissue structure. These observations could provide new insights into how cartilage and chondrocytes behave under the similar conditions found in OA.
